# Prognostic Factors of Gliosarcoma in the Real World: A Retrospective Cohort Study

**DOI:** 10.1155/2023/1553408

**Published:** 2023-01-30

**Authors:** Ziye Yu, Zhirui Zhou, Ming Xu, Kun Song, Jingjing Shen, Wenhao Zhu, Liqun Wei, Hongzhi Xu

**Affiliations:** ^1^Department of Neurosurgery, Huashan Hospital, Shanghai Medical College, Fudan University, 200040, China; ^2^National Center for Neurological Disorders, 200040, China; ^3^Shanghai Key Laboratory of Brain Function and Restoration and Neural Regeneration, 200040, China; ^4^Neurosurgical Institute of Fudan University, 200040, China; ^5^Shanghai Clinical Medical Center of Neurosurgery, 200040, China; ^6^Department of Radiotherapy, Huashan Hospital, Fudan University, 200040, China; ^7^Department of Anesthesiology, Huashan Hospital, Fudan University, 200040, China

## Abstract

**Purpose:**

Gliosarcoma is a histopathological variant of glioblastoma, which is characterized by a biphasic growth pattern consisting of glial and sarcoma components. Owing to its scarcity, data regarding the impact of available treatments on the clinical outcomes of gliosarcoma are inadequate. The purpose of this retrospective cohort study was to analyze the prognostic factors of gliosarcoma.

**Methods:**

By screening the clinical database of neurosurgical cases at a single center, patients with gliosarcoma diagnosed histologically from 2013 to 2021 were identified. Clinical, pathological, and molecular data were gathered founded on medical records and follow-up interviews. Prognostic factors were derived using the Cox proportional hazards model with backward stepwise regression analysis.

**Results:**

Forty-five GSM patients were included. Median overall survival was 25.6 months (95% CI 8.0–43.1), and median relapse-free survival was 15.2 months (95% CI 9.7–20.8). In multivariable analysis, total resection (*p* = 0.023, HR = 0.192, 95% CI 0.046–0.797) indicated an improved prognosis. And low expression of Ki-67 (*p* = 0.059, HR = 2.803, 95% CI 0.963–8.162) would be likely to show statistical significance. However, there might be no statistically significant survival benefit from radiotherapy with concurrent temozolomide (*n* = 33, 73.3%, log-rank *p* = 0.99) or adjuvant temozolomide (*n* = 32, 71.1%, log-rank *p* = 0.74).

**Conclusion:**

This single-center retrospective study with a limited cohort size has demonstrated the treatment of gross total resection and low expression of Ki-67 which are beneficial for patients with GSM, while radiotherapy or temozolomide is not.

## 1. Introduction

Gliosarcoma (GSM) is a rare malignant tumor of the central nervous system (CNS) and has been classified as glioblastoma (GBM) since the publication of 2016 World Health Organization Classification of Tumors of the Central Nervous System. It is considered as a histopathological variant of GBM, as well as epitheliod GBM and giant cell GBM, accounting for about 2-4% of all cases [1, 2]. GSM presents unique histopathological manifestations characterized by a biphasic growth model of glial and sarcomatous elements. The glioma components often exhibit the typical characteristics of glioblastoma and have different degrees of anaplasia and glial fibrillary acidic protein (GFAP) expression. Meanwhile, the sarcomatoid area appears microscopically which shows dense long-spindle cells arranged within a fishbone fibrosarcoma structure and occasionally malignant fibrous histiocytomas. The key points of diagnosis are the reticular fibres in sarcoma and GFAP expression in glioma [3]. GSM has similar radiological and clinical representations to GBM but a comparatively poorer prognosis [4–7]. Several researches have reported the median overall survival (OS) of gliosarcoma ranging from 6.6 to 18.5 months [8–11]. At present, special therapies for GSM are virgin, and treatments still cannot exceed the limits of GBM guidelines, including maximum surgical resection, radiotherapy (RT), and temozolomide (TMZ) [12].

Data regarding the impact of excision extension and postoperative adjuvant therapy on GSM outcomes are insufficient. Although gross total resection (GTR) of GBM independent of adjuvant therapy has been associated with progression-free survival (PFS) improvement, this has not yet been confirmed in GSM [11]. Some studies have elucidated a potential a possible benefit of RT and TMZ for GSM [10, 13]. However, there is still considerable controversy due to the lack of forward-looking clinical evidence [14, 15]. Distinctive histopathological properties may be associated with the differential therapeutic susceptibility between GSM and GBM. In addition, age, tumor size [16], and the diagnosis of primary or secondary [17] have also been reported being associated with GSM prognosis. There are still questions: what type of patient features indicates a better prognosis, and what kind of treatments can benefit GSM patients.

Here, we performed a retrospective cohort study of 45 GSM patients at single center. The purposes of our study were to analyze the independent prognostic factors of GSM and to understand the efficacy of classical treatments on survival outcomes.

## 2. Materials and Methods

### 2.1. Patients

**Figure 1:**
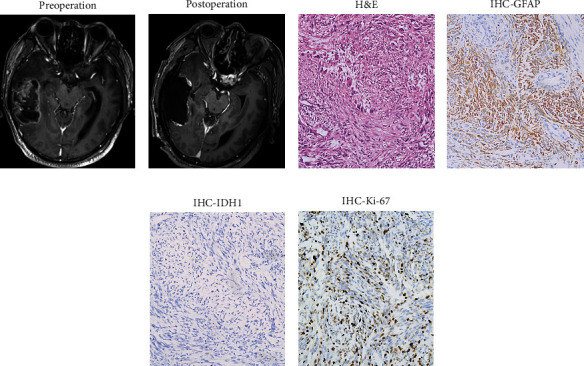
(a, b) Horizontal T1-weighted contrast-enhanced MRI imaging of GSM located in the temporal lobe. (c–f) Representative images of GSM stained using HE and immunolabeled using antibodies to GFAP, IDH1, and Ki-67. Through HE staining (c) and GFAP (d) staining, it can be observed in the biphasic growth pattern of GFAP-positive glioma components and GFAP-negative sarcoma components.

From November 2013 until May 2021, patients with pathologically authenticated GSM were scanned from the medical record database of our hospital. Below are other criteria for inclusion. Patients were treated with at least one surgical resection of GSM, including the primary operation and reoperation. In accordance with the safety principle, the focus and surrounding brain tissue should be removed as much as possible. Patients had undergone at least one postoperative cranial magnetic resonance (MR) or computerized tomography (CT) examination, which was compared with the preoperative imaging data to clarify the location and integrity of resection (Figures 1(a) and 1(b)). The GSM was diagnosed according to the biphasic growth pattern of hematoxylin-eosin (HE) staining as well as GFAP staining demonstrating GFAP-positive glioma components and GFAP-negative sarcoma components containing tumor spindle cells (Figures 1(c)–1(f)). The pathological specimens before the release of 2016 World Health Organization Classification of Tumors of the Central Nervous System were reviewed and confirmed by the pathologist. Radiologic and pathologic findings were read and examined by specialists who did not know patient information following the principle of blindness.

During the course of clinical data collection, we found that data collected since 2013 was relatively intact due to our electoral medical record system. Patients were routinely followed up every three months until May 2022. Meanwhile, there was no notable change in the treatment of GSM during this period. We excluded the patients with uncertain pathological diagnosis or severely incomplete clinical data. Following the identification of the cohort, the clinical records of patients in the cohort were reviewed carefully, including disease-specific demographic, clinical, and treatment characteristics.

### 2.2. Data Compilation

Clinical or treatment-related variables included age, sex, Karnofsky performance score (KPS), diagnosis of primary or secondary, single or multiple lesions, tumor location, preoperative size, epileptic seizure, intracranial hypertension, Ki-67 level, genetic mutation (e.g., IDH1, p53, PTEN, MGMT, 1p/19q codeletion, TERT, BRAF, PIK3CA, ATRX, and EGFR), and surgical resection extension, adjuvant therapies (RT, concurrent TMZ, adjuvant TMZ, and targeted therapy). The conditions of postoperative complications were difficult to collect and were not included in the analysis due to the high scarcity and the poor reliability. Tumor-specific characteristics were acquired from the initial MR or CT examinations. Typically, early imaging examinations provided a generalized diagnosis such as glioma instead of GSM. The final diagnosis was pathologically determined during subsequent surgical treatment.

### 2.3. Outcomes

We defined the first diagnosis date as the day of the first neurosurgery for GSM confirmed by postoperative pathology, and the OS was calculated from this day as the starting point.

In 8 cases of secondary GSM, all of these patients had explicit primary and secondary pathological diagnosis. The primary diagnosis was GBM (WHO IV) in 5 cases and non-GBM (WHO II-III) in 3 cases. The location of the secondary GSM was identical to the primary tumor, and the time interval between two onsets was no more than two years. According to 2016 World Health Organization Classification of Tumors of the Central Nervous System, these patients can be clearly defined as secondary GSM. Before the first diagnosis of GSM, all secondary GSM patients in this cohort had received neurosurgery and at least one adjuvant treatment. The diagnosis of primary and secondary is worth being included in the Cox proportional hazards model.

Another survival analysis index was relapse-free survival (RFS). For either primary or secondary GSM after tumor resection, relapse of the disease was defined as the date of radiographic recurrence necessitating either secondary surgical intervention or adjuvant therapy. RFS is defined from the date of the first resection of GSM lesions until the date of the first indication of tumor recurrence during the imaging examination. Clinical manifestations, multiple imaging examinations, and secondary postoperative pathology identified and excluded the possibility of tumor pseudoprogression in recurrent patients.

### 2.4. Statistical Analysis

The Cox regression model was used for univariable analysis, and the hazard ratio (HR) and its 95% confidence interval (95% CI) were estimated. Kaplan-Meier survival curves were plotted according to different prognostic factors and were analyzed using log-rank test. Then, multivariate analysis recruiting proper variables was performed by the backward regression method to confirm the statistical significance of prognostic factors. Calculations and graphical representations were performed using the *R* software package (version 4.0.3, The *R* Foundation for Statistical Computing).

## 3. Results

### 3.1. Cohort Characteristics

A total of 45 patients with histopathologically confirmed GSM between 2013 and 2021 were included (Table 1). As of the last follow-up, 30 patients (66.7%) had relapsed from the first GSM resection operation. Among them, 24 patients (53.3%) had passed away. In 15 patients (33.3%), no tumor recurrence was observed during postoperative imaging reexamination. In the cohort, 26 cases were medial and young (age<60, 57.8%), and 32 cases were males (71.1%). Most patients were diagnosed with primary GSM (*n* = 37, 82.2%), with the remainder being secondary GSM transformed from other types of glioma (*n* = 8, 17.8%). Median preoperative KPS was 80 (range 20-100). Preoperatively, 5 patients (11.1%) had epileptic seizures, and 36 patients (80%) suffered from ventricular enlargement and intracranial hypertension caused by tumor compression. Almost all but one patient had a single lesion (*n* = 44, 97.8%), primarily in temporal (*n* = 19, 42.2%) and frontal (*n* = 12, 26.7%) lobes. Other tumor locations included parietal lobe (*n* = 5, 11.1%), basal ganglia (*n* = 4, 8.9%), callosum (*n* = 3, 6.7%), thalamus (*n* = 1, 2.2%), and brainstem (*n* = 1, 2.2%). In terms of laterality, the left side (*n* = 20, 44.4%) was a little more than the right side (*n* = 16, 35.6%), and others were located deep in the brain (*n* = 9, 20.0%). Most tumors had a maximum diameter greater than 3 centimeters (*n* = 32, 71.1%).

The majority successfully underwent GTR (*n* = 41, 91.1%), and the remaining patients underwent subtotal resection (STR) and near total resection (NTR), based on the detailed surgical records and postoperative imaging data of each patient. Benefit from the progress of surgical technology and strictly observing surgical indications, the complete resection rate of malignant brain tumors increases gradually in our medical center, and some deep brain tumors can also be totally removed.

Except for one perioperative death, all cases had received at least one adjuvant therapy within the specified time, mainly RT (*n* = 33, 73.3%), concurrent TMZ (*n* = 33, 73.3%), and adjuvant TMZ (*n* = 32, 71.1%). This is the reliable information from clinical data. Some patients (*n* = 11) only accomplished adjuvant TMZ treatment because of terrible postoperative physical condition or poor tolerance to radiotherapy. Coincidentally, all patients receiving RT in this study took TMZ simultaneously, and part of them (*n* = 21) also received adjuvant TMZ. A few patients were also treated with nimustine (*n* = 4, 8.8%) and bevacizumab (*n* = 2, 4.4%). Some recidivist patients received reoperation (*n* = 12, 40%) or secondary radiotherapy (*n* = 7, 23.3%), and five of them received both of them.

In some patients, gene mutations were detected through next generation sequencing (NGS) and/or immunohistochemistry (IHC). The results are listed in Table 1.

### 3.2. Median OS and RFS

**Figure 2:**
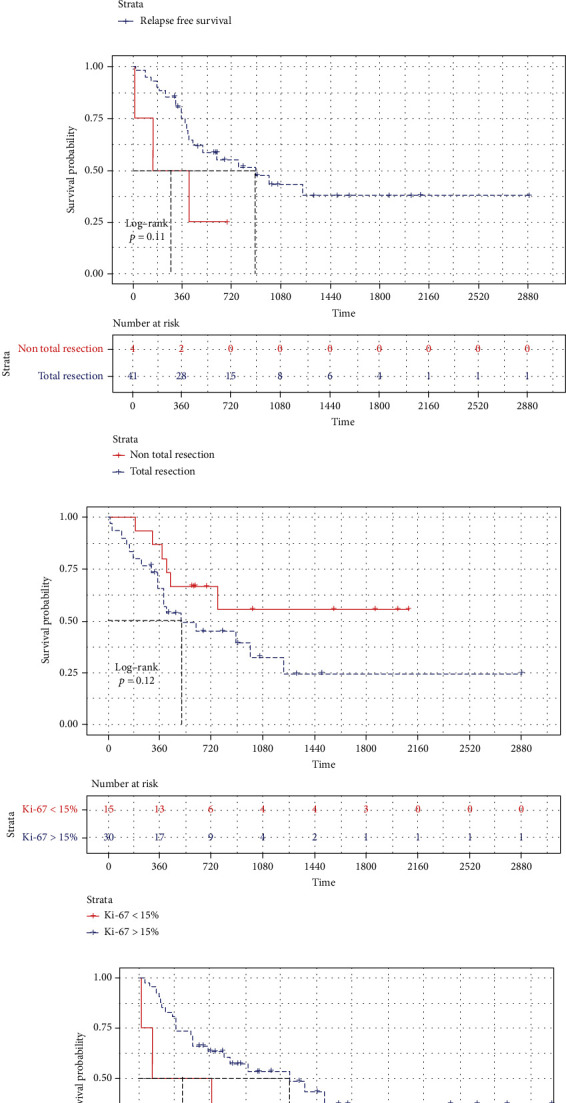
Kaplan-Meier curves for OS (a) and RFS (b) of all patients included in our cohort. (c) Kaplan-Meier curves and log-rank tests according to OS were used for analysis of prognostic factors. According to resection range, they were divided into total resection group and nontotal resection group. (d) According to the expression level of Ki-67, they were divided into high expression group and low expression group. (e, f) Kaplan-Meier curves and log-rank tests according to RFS were used for analysis of prognostic factors.

After discharge, patients were systematically reevaluated and reexaminated every three months. Most patients were given a long-term follow-up of more than 2 years, and the minimum follow-up period was 4 quarters. Survival data for 45 patients was clarified by April 2022, and the date of death and recurrence was precisely obtained. The median OS and the median RFS were calculated via the Kaplan-Meier curve. In this cohort, the median OS was 25.6 months (95% CI 8.0–43.1) (Figure 2(a)), and the median RFS was 15.2 months (95% CI 9.7–20.8) (Figure 2(b)).

### 3.3. Characteristics Associated with OS

Potential prognostic factors were analyzed using the Cox proportional hazards model (Table 2). In backward regression multivariable analysis excluding the variables of all gene mutations but p53 due to their incomplete data, total resection (*p* = 0.023, HR = 0.192, 95% CI 0.046–0.797) was significantly associated with longer OS, indicating a better prognosis of GSM patients. Moreover, expression level of Ki-67 (*p* = 0.059, HR = 2.803, 95% CI 0.963–8.162) would probably be statistically significant if the sample size increased (Table 2).

However, none of RT with concurrent TMZ (*p* = 0.991, HR = 0.995, 95% CI 0.389–2.546) and adjuvant TMZ (*p* = 0.742, HR = 0.860, 95% CI 0.351–2.110) indicated a statistical significance either in univariable and multivariable analysis (Table 2).

All patients with secondary GSM (*n* = 8) received postoperative RT or TMZ after the first operation. Of them, 4 patients received renewedly radiotherapy and concurrent TMZ after the second operation, and others also accepted adjuvant TMZ treatment. Compared with patients with primary GSM, there was no significant difference in patients with secondary GSM (*p* = 0.749, HR = 0.850, 95% CI 0.315–2.298), whether for GBM-origined (*p* = 0.317, HR = 1.742, 95% CI 0.588–5.166) or non-GBM-origined (*p* = 0.507, HR = 0.503, 95% CI 0.066–3.827) (Table 2).

**Figure 3:**
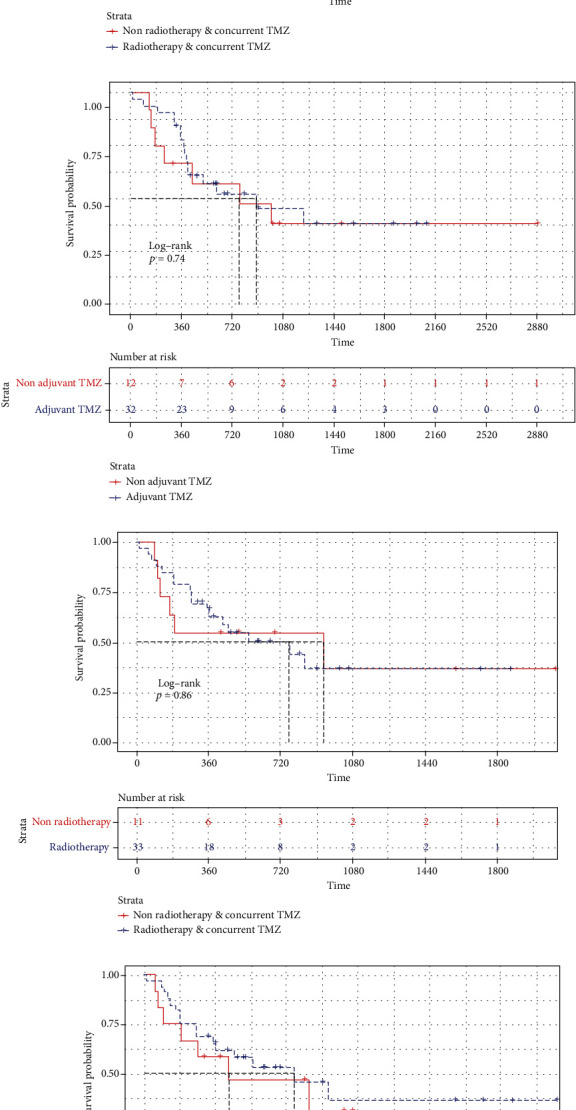
The results of Kaplan-Meier curves and log-rank tests showed that the effect of radiotherapy and concurrent TMZ (a) or adjuvant TMZ (b) on the OS benefits of GSM was not statistically significant. The results of Kaplan-Meier curves and log-rank tests according to RFS indicated that there was no statistically significant impact of radiotherapy and concurrent TMZ (c) or adjuvant TMZ (d).

Kaplan-Meier curves have been drawn, respectively, for interested factors, respectively. None of them demonstrated a significant difference. Median OS was 29.7 months (95% CI 11.5–47.9) in the total resection group (*n* = 41, 91.1%) and 4.8 months (95% CI 0.0–18.1) in the nontotal resection group (*n* = 4, 8.9%), *p* = 0.11 (Figure 2(c)). Median OS was 17.0 months (95% CI 8.0–43.1) in the Ki-67 high expression group (*Ki* − 67 > 15%, *n* = 30, 66.7%) and cannot be calculated in the Ki-67 low expression group (Ki − 67 ≤ 15%, *n* = 15, 33.3%) because the median OS is unreached, *p* = 0.12 (Figure 2(d)). Median OS was 25.6 months (95% CI 9.2–42.0) in the RT with the concurrent TMZ group (*n* = 33, 73.3%) and 41.0 months (95% CI 0.0–82.1) in the non-RT with the concurrent TMZ group (*n* = 11, 24.4%), *p* = 0.99 (Figure 3(a)) and 29.7 months (95% CI 4.1–55.3) in the adjuvant TMZ group (*n* = 32, 71.1%) and 25.6 months (95% CI 0.0–54.3) in the nonadjuvant TMZ group (*n* = 12, 26.7%), *p* = 0.74 (Figure 3(b)).

Median OS was 25.6 months (95% CI 9.2–42.0) in the RT with the concurrent TMZ group (*n* = 33, 73.3%) and 41.0 months (95% CI 0.0–82.1) in the non-RT with the concurrent TMZ group (*n* = 11, 24.4%), *p* = 0.99 (Figure 3(a)) and 29.7 months (95% CI 4.1–55.3) in the adjuvant TMZ group (*n* = 32, 71.1%) and 25.6 months (95% CI 0.0–54.3) in the nonadjuvant TMZ group (*n* = 12, 26.7%), *p* = 0.74 (Figure 3(b)).

In addition, according to the results of NGS and/or immunohistochemistry (IHC), the fact whether some gene loci were mutated was used as a prognostic factor for survival analysis, including p53 (negative, *n* = 14, 33.3% vs. positive, *n* = 28, 66.7%, log-rank *p* = 0.81), ATRX (negative, *n* = 8, 21.6% vs. positive, *n* = 29, 88.4%, log-rank *p* = 0.16), BRAF (negative, *n* = 19, 90.5% vs. positive, *n* = 2, 9.5%, log-rank *p* = 0.27), TERT (negative, *n* = 5, 27.8% vs. positive, *n* = 13, 72.2%, log-rank *p* = 0.025), MGMT methylated (negative, *n* = 11, 64.7% vs. positive, *n* = 6, 35.3%, log-rank *p* = 0.95), 1p/19q codeletion (negative, *n* = 10, 66.7% vs. positive, *n* = 5, 33.3%, log-rank *p* = 0.37), PIK3CA (negative, *n* = 9, 81.8% vs. positive, *n* = 2, 18.2%, log-rank *p* = 0.47), EGFR (negative, *n* = 8, 80.0% vs. positive, *n* = 2, 20.0%, log-rank *p* = 0.97), and PTEN (negative, *n* = 5, 21.6% vs. positive, *n* = 4, 88.4%, log-rank *p* = 0.81).

### 3.4. Characteristics Associated with RFS

We carried out multivariable analysis to calculate RFS using the same method and variables. Similar to OS benefits, the results showed that the variable of total resection (*p* = 0.022, HR = 0.181, 95% CI 0.042–0.782) and low expression of Ki-67 (*p* = 0.052, HR = 2.996, 95% CI 0.992–9.053) were helpful to improve RFS (Table 2).

Similarly, RT with concurrent TMZ (*p* = 0.861, HR = 0.919, 95% CI 0.360–2.348) and adjuvant TMZ (*p* = 0.631, HR = 0.804, 95% CI 0.330–1.959) did not demonstrate RFS benefits either in univariable and multivariable analysis (Table 2).

There was no distinct difference between the primary group and the secondary group (*p* = 0.715, HR = 1.202, 95% CI 0.448–3.228), whether for GBM-origined (*p* = 0.417, HR = 1.566, 95% CI 0.529–4.636) or non-GBM-origined (*p* = 0.646, HR = 0.624, 95% CI 0.083–4.684) (Table 2).

Kaplan-Meier curves were drawn, respectively, for interested factors, and none of them were considered significant that could affect RFS. Median RFS was 25.3 months (95% CI 9.5–41.1) in the total resection group (*n* = 41, 91.1%) and 2.3 months (95% CI 0.0–13.9) in the nontotal resection group (*n* = 4, 8.9%), *p* = 0.12 (Figure 2(e)). Median RFS was 18.5 months (95% CI 6.0–31.0) in the Ki-67 high expression group (Ki − 67 > 15%, *n* = 30, 66.7%) and cannot be counted in the Ki-67 low expression group (Ki − 67 ≤ 15%, *n* = 15, 33.3%) because the death toll is less than half, *p* = 0.12 (Figure 2(f)).

Median RFS was 25.3 months (95% CI 10.4–40.2) in the RT with the concurrent TMZ group (*n* = 33, 73.3%) and 31.1 months (95% CI 0.0–64.1) in the non-RT with the concurrent TMZ group (*n* = 11, 24.4%), *p* = 0.86 (Figure 3(c)) and 25.3 months (95% CI 9.7–40.9) in the adjuvant TMZ group (*n* = 32, 71.1%) and 14.3 months (95% CI 0.0–35.2) in the nonadjuvant TMZ group (*n* = 12, 26.7%), *p* = 0.63 (Figure 3(d)).

## 4. Discussion

As a histological variant of GBM, GSM has some different prognostic factors. Reviewing the clinical data of the cohort, the people with high incidence of GSM are concentrated around the age of 60 (50 ≤ age < 70, *n* = 29, 64.4%), mainly male (*n* = 32, 71.1%) and showing a predilection for temporal lobe (*n* = 19, 42.2%). The situation is generally consistent with the previous research results [17–22]. In our cohort, the proportion of patients with secondary GSM is 17.8%, which is still consistent with existing reports [22, 23].

The median OS in our study was 25.6 months and longer than those in most other studies, which range from 5.7 months to 24.7 months [24, 25]. The results of our study suggested that total resection of GSM was associated with prolonged OS and RFS. Although some retrospective studies have reached the opposite conclusions [9, 11, 24], the resection range is a well-accepted independent prognostic factor for improved OS in GSM [4, 5, 17, 26, 27]. Cachia et al. reported that patients of primary GSM undergoing GTR tended to have a greater OS (median 24.7 months) than those having subtotal resection (median 10.1 months) [25]. And with advances in medical technology, more thorough surgeries were accepted by patients over the past six years. In our cohort, even if the lesions were deep in 9 patients, 41 patients had undergone GTR, and no patients received biopsy. Most patients were in relatively good physical condition at the beginning of treatment (median KPS was 80) and were more inclined to tolerate the treatments with large physiological burden.

Currently, almost GSM patients are guided to accept the Stupp protocol in clinical practice, RT, and chemotherapy following surgery as same as GBM [19]. Radiotherapy has been suggested to improve the outcomes of patients, because it may increase OS by 2-4 months [28]. TMZ has been proved as the most effective chemotherapeutic drug for high-grade gliomas [9, 29]. But due to the lack of prospective studies or large-scale multicenter retrospective studies, the debate on the therapeutic value of RT and TMZ in GSM remains unresolved [5]. The scholars from Stanford University Medical Center provided that a significantly improved PFS (median 32.97 months) and OS (median 56.73 months) occurred in patients receiving surgical resection followed by RT and concurrent TMZ [17]. Different from their results, RT, concurrent TMZ, and adjuvant TMZ did not show any survival benefits. Our study is inclined to support the view that RT or TMZ is ineffective for GSM patients. The histopathological differences between GSM and classical GBM cannot be overlooked. GSM is rich in sarcoma components, which were insensitive to RT and chemotherapy [30], which is related to minimal statistical effectiveness. In addition, lots of patients in this cohort have not not yet arrived at the terminal of the life train, which inevitably influences the judgment of the effect of RT and chemotherapy.

The lower Ki-67 expression is parallel to a lower rate of tumor cell division [31], which indicates a longer RFS. This inference has been supported in the study of GSM [32], and our results enhance the reliability.

In univariable analysis, no statistically significant parameters were found (*p* ≤ 0.05) but lesion number, but there is only one case with multiple lesions. The enormous sample size between groups makes the results lack credibility and be unable to participate in multivariable analysis. Existing reports suggest that TERT [33] and PIK3CA [34] mutations are frequent in GSM, but there is still a lack of evidence of prognostic relevance. In our study, the *p* values of TERT are below 0.05 in the log-rank test. The small sample size determines that the univariable analysis of these transgenations has no practical reference value. The results are just listed.

A series of other favorable prognostic factors have been reported previously, including MGMT methylation [5, 14], tumor size [21, 35], temporal tumor location [36], and younger age [5, 13, 21, 32, 35]. These conditions are rarely repeated because of small sample size. No such association was seen in our study.

Compared to prospective studies or large multicenter randomized controlled trials (RCTs), there are many limitations to the single-center retrospective cohort studies for rare diseases. The limited cohort size limits the analysis of potential subset analysis and may hinder our ability to adequately adjust for confounding covariates. All patients had obtained postoperative pathological diagnosis when they were selected into the cohort, which might result in inevitable selection bias. We should be cautious about the conclusions of this study, but we are confident to persist in the clinical value of our results for further prospective study or RCTs.

## 5. Conclusions

This study is a retrospective cohort study with limited cohort size, our results demonstrate that GTR and low expression of Ki-67 are of great significance to the prognosis of GSM, while the survival benefits of adjuvant therapies including RT or TMZ are limited.

## Figures and Tables

**(a):** 

Characteristics	Number of patients (*n* = 45)	Proportion (%)
Relapse	30	66.7
Death	24	53.3
Age		
Average age, years	53.2	
Median age, years	56	
<60	26	57.8
≥60	19	42.2
Sex		
Male	32	71.1
Female	13	28.9
KPS		
≤70	15	33.3
>70	30	66.7
Primary or secondary		
Primary GSM	37	82.2
Secondary GSM	8	17.8
GBM-origined	5	11.1
Non-GBM-origined	3	6.6
Number of lesions		
Single	44	97.8
Multiple	1	2.2
Size, diameter		
≤3 cm	13	28.9
>3 cm	32	71.1
Location		
Temporal	19	42.2
Frontal	12	26.7
Parietal	5	11.1
Basal ganglia	4	8.9
Callosum	3	6.7
Thalamus	1	2.2
Brainstem	1	2.2
Laterality		
Left	20	44.4
Right	16	35.6
Profound	9	20.0
Epileptic seizure		
No	40	88.9
Yes	5	11.1
Intracranial hypertension		
No	9	20.0
Yes	36	80.0
Level of Ki-67		
≤15%	15	33.3
>15%	30	66.7
Resection range		
GTR	41	91.1
STR/NTR	4	8.9
Biopsy	0	0
Radiotherapy		
No	11	24.4
Yes	33	73.3
Missing	1	2.2
Dose, mean (SD)	57.97 (2.75)	
Concurrent TMZ		
No	11	24.4
Yes	33	73.3
Missing	1	2.2
Adjuvant TMZ		
No	12	26.7
Yes	32	71.1
Missing	1	2.2

**(b):** 

Gene locus	Wild-type (%)	Mutated (%)	Missing
IDH1	40 (100.0)	0 (0.0)	5
p53	14 (33.3)	28 (66.7)	3
PTEN	5 (55.6)	4 (44.4)	36
MGMT	11 (64.7)	6 (35.3)	28
1p/19q codeletion	10 (66.7)	5 (33.3)	30
TERT	5 (27.8)	13 (72.2)	27
BRAF	19 (90.5)	2 (9.5)	24
PIK3CA	9 (81.8)	2 (18.2)	34
ATRX	8 (21.6)	29 (78.4)	8
EGFR	8 (80.0)	2 (20.0)	35

**(a):** 

Variable name	Univariable analysis, OS		Multivariable analysis, OS	
	*p* value	HR (95% CI)	*p* value	HR (95% CI)
Age	0.732	1.151 (0.515-2.572)		
Sex	0.358	0.678 (0.296-1.553)		
Primary or secondary	0.749	0.850 (0.315-2.298)		
Primary vs. GBM-origined	0.317	1.742 (0.588–5.166)		
Primary vs. non-GBM-origined	0.507	0.503 (0.066-3.827)		
KPS	0.847	0.919 (0.392–2.157)		
Location	0.548			
Frontal vs. deep	0.195	0.452 (0.136-1.500)		
Parietal vs. deep	0.989	0.990 (0.244-4.024)		
Temporal vs. deep	0.364	0.624 (0.226-1.728)		
Size	0.334	0.657 (0.280-1.542)		
Number of lesions	0.036	0.095 (0.011-0.853)		
Epileptic seizure	0.684	0.739 (0.172-3.170)		
Intracranial hypertension	0.377	0.639 (0.237-1.725)		
Ki-67 level	0.132	2.039 (0.807-5.151)	0.059	2.803 (0.963-8.162)
Resection range	0.125	0.382 (0.111-1.306)	0.023	0.192 (0.046-0.797)
Radiotherapy	0.991	0.995 (0.389-2.546)		
Concurrent TMZ	0.991	0.995 (0.389-2.546)		
Adjuvant TMZ	0.742	0.860 (0.351-2.110)		
Gene mutation				
IDH1	—	—		
p53	0.807	0.893 (0.360-2.216)		
PTEN	0.809	1.221 (0.243-6.122)		
MGMT	0.953	0.950 (0.173-5.229)		
1p/19q codeletion	0.381	2.057 (0.410-10.318)		
TERT	0.209	47.014 (0.116-18994)		
BRAF	0.288	2.436 (0.472-12.569)		
PIK3CA	0.483	2.262 (0.232-22.095)		
ATRX	0.165	0.483 (0.173-1.349)		
EGFR	0.975	0.966 (0.111-8.415)		

**(b):** 

Variable name	Univariable analysis, RFS		Multivariable analysis, RFS	
	*p* value	HR (95% CI)	*p* value	HR (95% CI)
Age	0.700	1.171 (0.524-2.616)		
Sex	0.568	0.784 (0.341-1.805)		
Primary or secondary	0.715	1.202 (0.448-3.228)		
Primary vs. GBM-origined	0.417	1.566 (0.529-4.636)		
Primary vs. non-GBM-origined	0.646	0.624 (0.083-4.684)		
KPS	0.800	0.896 (0.383-2.096)		
Location	0.708			
Frontal vs. deep	0.367	0.578 (0.176-1.900)		
Parietal vs. deep	0.995	1.004 (0.250-4.041)		
Temporal vs. deep	0.424	0.660 (0.238-1.831)		
Size	0.426	0.706 (0.300-1.663)		
Number of lesions	0.022	0.071 (0.007-0.679)		
Epileptic seizure	0.857	0.875 (0.205-3.731)		
Intracranial hypertension	0.513	0.714 (0.260-1.958)		
Ki-67 level	0.130	2.066 (0.809-5.278)	0.052	2.996 (0.992-9.053)
Resection range	0.137	0.395 (0.116-1.345)	0.022	0.181 (0.042-0.782)
Radiotherapy	0.861	0.919 (0.360-2.348)		
Concurrent TMZ	0.861	0.919 (0.360-2.348)		
Adjuvant TMZ	0.631	0.804 (0.330-1.959)		
Gene mutation				
IDH1	—	—		
p53	0.995	0.997 (0.402-2.474)		
PTEN	0.745	1.307 (0.261-6.550)		
MGMT	0.992	1.009 (0.184-5.527)		
1p/19q codeletion	0.433	1.900 (0.382-9.440)		
TERT	0.202	49.123 (0.125-19.370)		
BRAF	0.270	2.529 (0.487-13.134)		
PIK3CA	0.838	1.252 (0.145-10.817)		
ATRX	0.298	0.581 (0.209-1.617)		
EGFR	0.951	1.070 (0.124-9.249)		

## Data Availability

The datasets used and/or analyzed during the current study are available from the corresponding author on reasonable request.
